# Efficacy and safety of use of ultrasound enhancing agent in patients hospitalized with COVID-19

**DOI:** 10.1007/s10554-023-03032-4

**Published:** 2023-12-14

**Authors:** Gabriel Bioh, Christina Botrous, Roxy Senior

**Affiliations:** 1 Department of Cardiology, Northwick Park Hospital, Harrow, UK; 2 Department of Cardiology, Royal Brompton Hospital, London, SW3 6NP UK; 3 National Heart and Lung Institute, Imperial College, London, UK; 4 Department of Cardiology, Royal Brompton Hospital and Imperial College London, London, UK

**Keywords:** COVID-19, Echocardiography, Ultrasound enhancing agent

## Abstract

**Purpose:**

The efficacy and safety of ultrasound enhancing agent (UEA) was unknown in the COVID-19 hospitalized patients. We set out to establish the utility of UEA and its safety profile.

**Methods:**

A retrospective observational study of prospectively assessed hospitalized patients referred for transthoracic echocardiography (TTE) for suspected cardiac pathology due to COVID-19. The indications and subsequent ability to answer the indications for all TTE were reviewed, as well as impact on diagnosis and management. UEA safety was considered through 48 h mortality.

**Results:**

From a total of 364 patients (mean age 64.8yrs, 64% males) hospitalized with COVID-19 with TTE requested, an indication could be identified in 363, and 61 required administration of UEA. Standard TTE was able to answer the original indication in 275 (75.8%) patients. This was increased to 322 (88.7%) patients, a relative increase of 17.1%, with the use of UEA (p < 0.001). There was subsequent change in diagnosis in 22 out of 61 (36%) patients receiving UEA and change in management in 13 out of 61 (21.3%). There was no significant increase in 48 h (p = 0.14) mortality with UEA use. The patient population of TTE with UEA versus TTE without UEA differed in having a higher incidence of left ventricular systolic dysfunction, right ventricular dilatation, and self-defined white ethnicity.

**Conclusion:**

The use of UEA in COVID-19 hospitalized patients, including those who were critically ill, provided incremental information when compared to TTE without UEA resulting in both changes in diagnosis and management plan and appears to be safe.

## Introduction

Early in the COVID-19 pandemic, many adaptations had to be made to the daily practice of cardiology with regard to infection control. To mitigate the spread of infection, use of cardiac investigations was limited, particularly within the cardiac imaging field due to the risks of cross-infection as well as utilisation of, at the time, limited supplies of personal protective equipment (PPE). With regard to echocardiography, given the need for close proximity to the patient, guidance was issued to limit scans to those absolutely clinically necessary to alter management as well as the need to abbreviate the scan to reduce potential exposure to COVID-19 [1–3]. Further to this, strict rules were implemented within our department mandating the disinfection of the entire Echo machine between patients to reduce the risk of cross infection.

COVID-19 posed considerable challenges for echocardiogram acquisition. Wearing full PPE whilst scanning, donning and doffing between patients and repeated disinfection of the Echo machine was physically demanding. Optimal positioning of the patient was often not practical due to their being acutely unwell and significantly short of breath. Many patients were ventilated on the intensive therapy unit (ITU), at times prone. So the need for ultrasound enhancing agent (UEA) to aid in the answering of the most fundamental of questions such as left ventricular ejection fraction (LVEF) and presence of regional wall motion abnormality (RWMA), as per international guidance [4, 5], was never more so needed.

However, with little known about the pathogenesis and natural history of COVID-19, early guidance was to avoid using UEA in COVID-19 patients with circulatory instability or critically ill [3]. There is a long history of caution surrounding the use of UEA. Early reports of deaths in 4 patients as well as around 190 adverse cardiopulmonary reactions, with temporal relationship to UEA use, led the US Food and Drug Administration (FDA) to issue a black box warning in October 2007, restricting use of the available UEAs at the time [6]. This prompted multiple studies in the following years establishing an evidence base for the safe use of UEA in patients including those in an outpatient setting, hospitalized and critically ill patients [7–10].

It is within this context that we sought to investigate the efficacy and safety of UEA use in the patients admitted to a UK National Health Service (NHS) hospital.

## Materials and methods

### Study population

Data was collected prospectively as part of a service evaluation of 364 admissions to the London North West University Healthcare NHS Trust referred for transthoracic echocardiography for suspected cardiac pathology due to COVID-19 from March 1st 2020 to February 28th 2021. However, we retrospectively collected data regarding UEA use. The diagnosis of COVID-19 was made based on symptoms suggestive of COVID-19 as well as a positive polymerase chain reaction (PCR) swab or a chest radiograph or a computed tomography (CT) scan of the chest demonstrating probable COVID-19. All included patients were clinically managed as COVID-19 infected with 338 PCR positive, and 26 negative but with radiographic evidence of COVID-19 infection.

### Data collection

The data was collected as part of a service evaluation reviewed by Hospital Research and development for which individual consent was not required for inclusion of data in the study as all data collected was routine clinical data.

#### Clinical data

This included patient demographics, blood biomarkers (1st Troponin, NTPro-BNP, D-dimer and Ferritin), comorbidities (hypertension, diabetes, cardiovascular disease, cerebrovascular disease, cancer, chronic kidney disease and respiratory disease), admission haemodynamics and mechanical ventilation use.

#### Echocardiographic data

All TTEs were performed at the bedside i.e. portable. Physicians and UEA trained cardiac physiologists carried UEA with them in anticipation of poor endocardial definition, non-UEA trained cardiac physiologists did not.

Personal protective equipment (PPE) was sourced and provided by the Northwick Park Cardiac research charity where Hospital Trust resources were insufficient hence ensuring continuity of TTE delivery. Full PPE- FFP3 mask, surgical gown, gloves, full face shield and hair covering- was worn for each patient, donning and doffing between patients unless in the same ward bay. The TTE equipment was, however, thoroughly cleaned with alcohol wipes between each patient.

TTEs were abbreviated to include the parasternal long-axis (PLAX) view, deep PLAX for pleural effusion, PLAX with colour flow (CF) through aortic valve (AV)and mitral valve (MV). The parasternal short-axis (PSAX) view was taken at the basal (for pulmonary measures), mid and apical level. The apical 4 chamber (A4C) view was taken with CF and doppler [pulse wave (PW) and continuous wave (CW)] through MV, tissue doppler imaging(TDI) [lateral, septal and right ventricle (RV)] and CF and CW through the tricuspid valve (TV). A focussed RV view was obtained for RV dimensions and function. An apical 5 chamber view was obtained if an abnormality of the aortic valve was seen from prior views, using CF, CW and PW. Finally, the apical 2 chamber (A2C), apical 3 chamber (A3C), subcostal 4 chamber and subcostal short-axis for inferior vena cava were obtained. Where contrast was required, we obtained an A4C with LV zoom, A2C with LV zoom, and A3C with LV zoom.

The echocardiographic parameters recorded for the study were left ventricular (LV) dimensional and functional measures, right ventricular (RV) dimensional and functional measures and pulmonary pressure measures. These data were used to identify significant differences between the TTE with UEA patients versus TTE without UEA patients that could account for different outcomes of the two groups apart from the most obvious difference of receiving UEA and not. The details of the specific measures taken are described in a previous publication [11] and documented in Tables 1 and 2 below.

#### UEA data

Firstly, the indications for all echocardiograms were recorded from the echo request directly with inpatient notes review where there was ambiguity. The outcomes of the echocardiograms were then recorded and whether this answered the original indication was determined. Further, the use of UEA was noted and whether this was able to answer the question posed by the original indication.

The inpatient notes were then reviewed in order to assess the effect of the echocardiogram on patient management by the clinical team responsible for the patient. The five points assessed were whether the indication for the test was answered, the diagnosis of the patient, choice of investigations, change to medication and change to anticoagulation - the latter three constitute change in management.

With regard to the safety of UEA, mortality within 24 and 48 h of first standard transthoracic echocardiogram (TTE) or first TTE with UEA was recorded. Patient mortality was determined from the first TTE of patients who did not then go on to have UEA. If the patient went on to have UEA, their first TTE, if not done at the same time, was not included in the time interval to avoid double counting the same patients.

### Statistical analysis

The IBM SPSS statistics 27 package was used for all statistical analysis. A P value of less than 0.05 was considered statistically significant. Student’s t test was used for comparison of means for normally distributed continuous data whilst the Mann-Whitney U Test used for non-normally distributed continuous data. Test for normality was by visual assessment of quantile-quantile plots and the Shapiro-Wilk test.

To assess the utility of UEA over that of standard TTE alone, McNemars test was used. The Chi-Square Test of Independence was used for the analysis of independent categorical data. However, Fisher’s Exact Test was used where the sample size was small (< 40) or the expected frequencies were less than 5 in 20% or more of cells.

Logistic regression was used to determine association with UEA use versus no UEA use. Only significant variables (p < 0.05), on univariable analysis, were entered into a multivariable analysis using the backward elimination method.

## Results

Of the 364 patients assessed, 233 patients were male (64.0%) and 131 female (36.0%). The mean age at admission was 64.8 years. The co-morbidities of these patients are shown in Table 1. Of these patients 61 (16.8%) received UEA.

**Table 1:** Table of recorded variables for Demographics, Blood work, Comorbid conditions, Haemodynamics and Ventilation. The variables were compared between the ultrasound enhancing agent (UEA) and no UEA population. Means and standard deviations or medians and interquartile ranges displayed for continuous variables and number and percentage for categorical variables. Number in square bracket records total number of patients. Significant p values are in bold

Variable	All patients (364)	No UEA (303)	UEA (61)	P value
**Demographics**				
Age at admission, years, mean (± SD)	64.8 (± 14.6) [364]	64.4 (± 15.1) [303]	66.5 (± 11.8) [61]	0.24
Gender, male, number (%)	233 (64%) [364]	189 (62%) [303]	44 (72%) [61]	0.15
Ethnicity, white, number (%)	78 (25%) [315]	57 (22%) [258]	21 (37%) [57]	**0.02**
BMI (kg/m2), mean, (± SD)	28.3 (± 7.2) [352]	28.3 (± 7.1) [292]	28.2 (± 8.0) [60]	0.70
**Co-morbid conditions**				
Hypertension, number (%)	203 (56%) [364]	168 (55%) [303]	35 (57%) [61]	0.78
Diabetes, number (%)	152 (42%) [364]	127 (42%) [303]	25 (41%) [61]	0.89
Cardiovascular disease, number (%)	90 (25%) [364]	72 (24%) [303]	18 (30%) [61]	0.34
Cerebrovascular disease, number (%)	43 (12%) [364]	37 (12%) [303]	6 (10%) [61]	0.60
Cancer, number (%)	35 (10%) [364]	26 (9%) [303]	9 (15%) [61]	0.14
Chronic Kidney Disease, number (%)	48 (13%) [364]	42 (14%) [303]	6 (10%) [61]	0.40
Respiratory disease, number (%)	91 (25%) [364]	76 (25%) [303]	15 (25%) [61]	0.94
**Blood biomarkers**				
1st Troponin T (ng/L) (normal range 0–15), median (IQR)	22 (44) [289]	22 (46) [240]	24 (42) [49]	0.67
1st NT-Pro BNP (ng/L), median (IQR)	957 (3453) [221]	1014 (4500) [178]	717 (1596) [43]	0.28
1st D-dimer (ug/L) (normal range 0.0–500), median (IQR)	1950 (7760) [263]	2120 (8540) [215]	1310 (3512) [48]	0.15
1st Ferritin (ug/L) (normal range 30–400), median (IQR)	816 (1126) [262]	869 (1230) [216]	759 (1056) [46]	0.45
**Haemodynamics prior to echocardiogram**				
Admission Heart Rate (beats per minute), mean (± SD)	101 (± 22.8) [193]	100 (± 23.4) [168]	107 (± 17.7) [25]	0.07
Admission Respiratory Rate (breaths per minute), mean (± SD)	31 (± 9.8) [191]	31 (± 10.0) [167]	30 (± 8.1) [24]	0.71
Admission Saturations on air (%), mean (± SD)	90 (± 10.8) [193]	90 (± 11.1) [168]	91 (± 9.0) [25]	0.72
Admission Systolic blood pressure, mmHg, mean (± SD)	136 (± 24.7) [190]	135 (± 23.9) [167]	142 (± 29.7) [23]	0.29
**Ventilation**				
Ventilated patients, number (%)	152 (42%) [363]	126 (42%) [302]	26 (43%) [61]	0.90

### UEA utility over standard transthoracic echocardiography

Of the 364 patients who underwent TTE in the study period of 1st March 2020 to 28 February 2021, the indication for echocardiogram could not be found for 1 patient.

The indications for TTE fell into the categories of (1) LV function assessment, (2) LV regional wall motion assessment, (3) RV function assessment, (4) Assessment of thrombus and (5) Pulmonary pressure assessment. Use of UEA was for left ventricular opacification and endocardial definition as well as right ventricular opacification and endocardial definition. On one occasion, UEA was utilized for perfusion assessment.

**Fig. 1:**
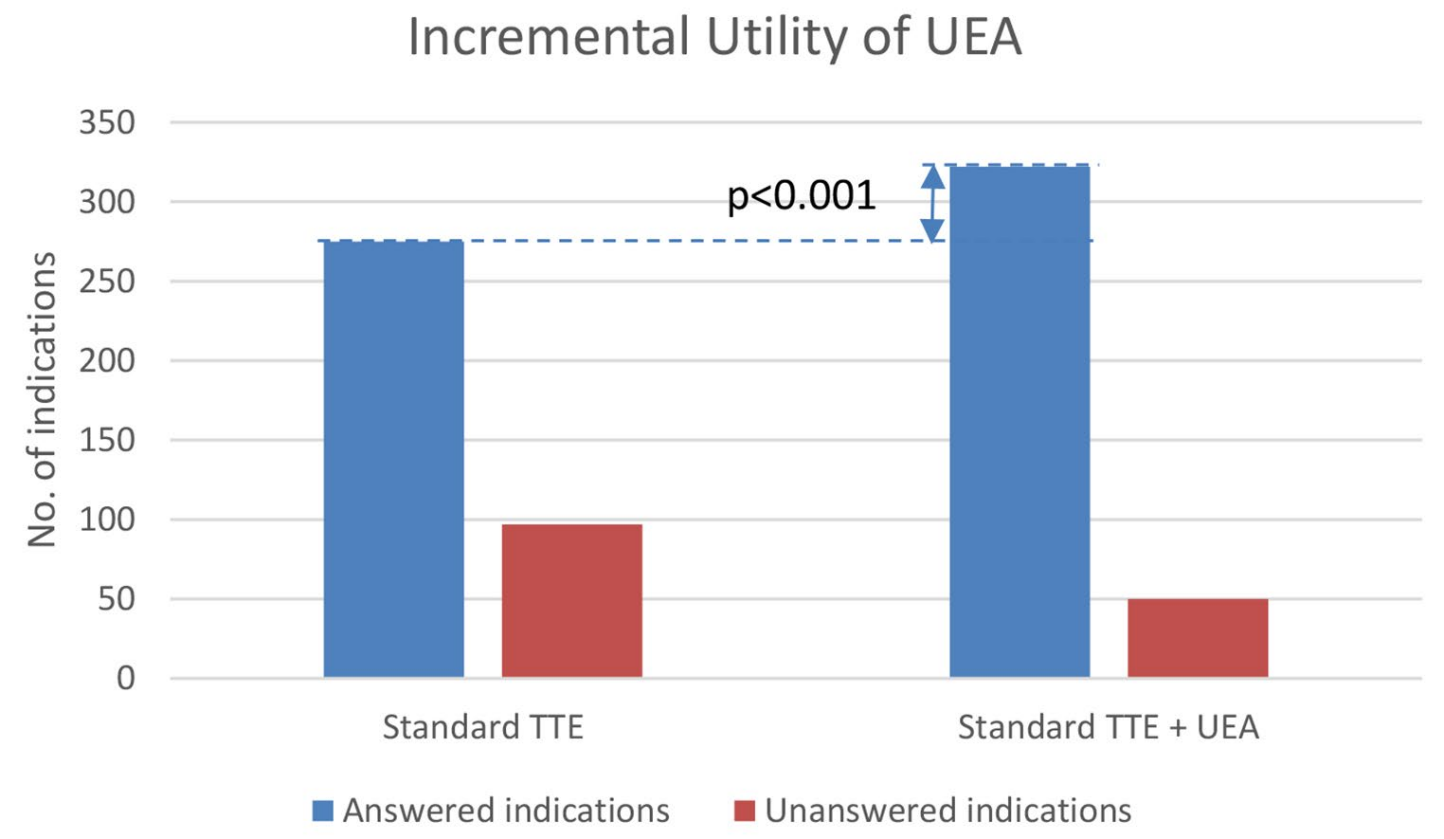
Incremental utility of UEA. Comparison of number of answered indications vs. unanswered indications for standard transthoracic echocardiography (TTE) alone vs. standard TTE followed by UEA. Number of answered indications increased from 275 to 322 when comparing Standard TTE vs. Standard TTE + UEA, p < 0.001

Standard TTE answered the indication in 275 (75.7%) patients. Of the remaining 88, UEA was used in 52 patients to answer the original indication. Of these, UEA facilitated answer in 47 (90.3%) giving an overall answer to original indication in 322 patients (88.7%), a relative increase of 17.1%, p < 0.001 (Fig. 1).

Of 9 patients where the original indication was answered by standard TTE, a further indication for UEA was raised, of whom 8 (88.9%) had an answer following UEA. Thus, overall, 61 patients received UEA and an answer was obtained in 55 patients (90.2%).

**Fig. 2:**
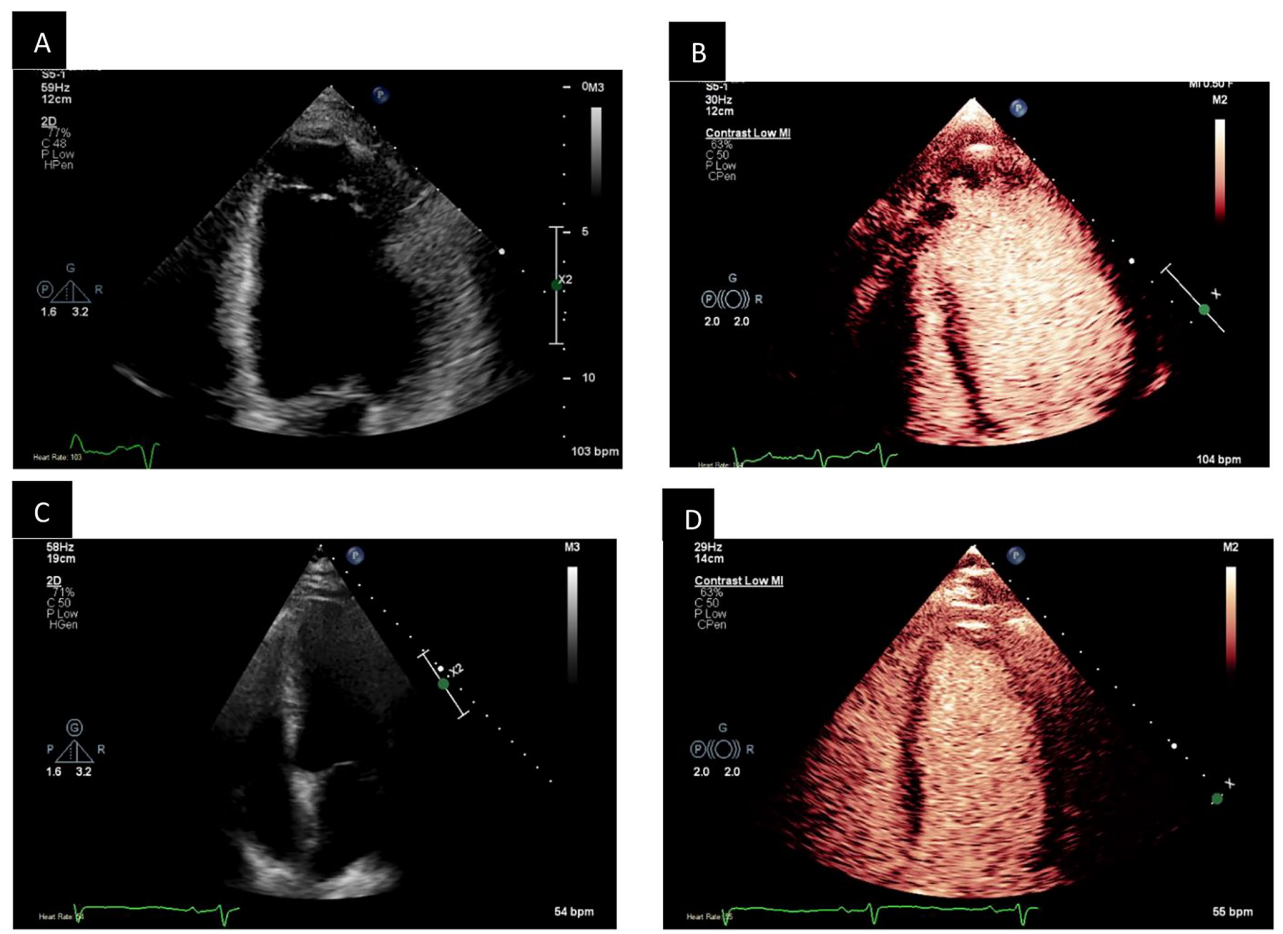
Examples of UEA utility (A) Apical 4 chamber view of a patient with akinetic apex with possible false tendons. Apical thrombus could not be excluded. (B) UEA demonstrates the presence of two apical thrombi. (C) Apical 4 chamber view of a patient with poor endocardial definition. Left ventricular ejection fraction could not be established. (D) UEA clearly defined the endocardial border leading to a diagnosis of globally reduced ejection fraction, 48% by Simpson’s biplane

Figure 2 shows examples of how UEA was able to answer the question raised in the cases of possible apical LV thrombus and LVEF assessment. Of the 61 patients who underwent UEA, 16 had SonoVue, 40 Luminity and in 5 patients the UEA type was not specified.

### Changes to diagnosis

UEA resulted in an alteration in diagnosis in 22 of 61 patients (36%). This was made up of a new diagnosis in 11 of 61 patients (18.0%) (heart failure with reduced LVEF in 6 patients, heart failure with RWMA in 1 patient, RV dysfunction in 2 patients, RV thrombus in 1 patient and LV thrombus in 1 patient). There was also revision of working diagnosis in 11 of 61 patients (18.0%) (reduced likelihood of acute coronary syndrome (ACS) in 4 patients, exclusion of heart failure with reduced LVEF in 6 patients and exclusion of RV apical thrombus in 1 patient).

### Changes in management of patients

#### Change in medications

There was a change in medication following UEA in 9 of 61 (14.8%) patients in the form of changes to anticoagulation (based on the presence or absence of thrombus) in 4 patients, commencing treatment for new heart failure with reduced LV ejection fraction for 2 patients, commencing ACS treatment in a patient, and stopping ACS medication in 2 patients where normal LV function and no RWMA were demonstrated (where clinical interpretation determined ACS unlikely, lack of RWMA and normal LV function were used to corroborate that interpretation).

#### Change in investigation

Changed investigation strategy was observed in 6 of 61 patients (9.8%). Changes to investigation included indication for inpatient coronary angiography in 1 patient, delay of coronary angiography to outpatient in 1 patient, cancellation of planned coronary angiography in 3 patients and indication for CT pulmonary angiography in 1 patient.

### Timing of UEA administration

UEA was given at the time of TTE in 33 of 61 patients (54.1%). The use of UEA necessitated a longer scan by 5 min on average. However, in 28 (45.9%) patients, a second visit to the patient was required to perform TTE with UEA as the sonographer was not trained to use UEA, the sonographer was trained but did not have any UEA or subsequent review of the TTE gave UEA indication. This second visit was a median of 3 days later.

### Demography, co-morbidity and echocardiographic characteristics of patients undergoing UEA versus no UEA

When comparing the demographics of patients in whom UEA was used compared to no UEA, there was no difference between age, gender and body mass index (BMI) but proportionately more patients with UEA were white versus non-white, p = 0.02. There was a trend toward higher BMI in the white group p = 0.06 as well as respiratory disease p = 0.10.

There was no difference between UEA use and no UEA use when comparing blood biomarkers, comorbidity, haemodynamics and use of mechanical ventilation except there was a trend towards higher heart rate in the UEA group (p = 0.07). The findings are summarized in Table 1.

Further comparison was made between the UEA use versus no UEA use populations with regard to LV, RV and pulmonary echocardiographic parameters (Table 2). Of the LV parameters, new LV systolic dysfunction was significantly higher in the UEA population, p = 0.01, with trend towards larger end-diastolic (p = 0.08) and end-systolic dimensions (p = 0.09). Of the RV parameters, presence of RV dilatation (≥ 4.1 cm) was significantly higher in the UEA population, p = 0.047, with a trend towards greater degree of RV dysfunction suggested by reduced TAPSE values (p = 0.05). There was no significant difference in the pulmonary parameters.

**Table 2:** Table of recorded variables for Left ventricle, Right ventricle and Pulmonary pressure. The variables were compared between the ultrasound enhancing agent (UEA) and no UEA population. Means and standard deviations are displayed for continuous variables and number and percentage for categorical variables. Number in square bracket records total number of patients. Significant p values are in bold

Variable	No UEA (303)	UEA (61)	P value
**Left ventricle**			
New LV systolic dysfunction, number (%)	27 (9%) [303]	12 (20%) [60]	**0.01**
LV end-systolic dimension (cm), mean (± SD)	2.9 (± 0.6) [298]	3.2 (± 0.9) [56]	0.09
LV end-diastolic dimension (cm), mean (± SD)	4.3 (± 0.7) [298]	4.5 (± 0.8) [57]	0.08
LA vol index (ml/m²), mean (± SD)	31.2 (± 15.5) [193]	29.9 (± 11.9) [41]	0.96
New elevated LV filling pressure + Normal LVEF > 50%, number (%)	12 (6%) [196]	4 (9%) [44]	0.50
New LV RWMA, number (%)	17 (7%) [231]	3 (7%) [43]	1.00
**Right Ventricle**			
New RV impairment by visual and numerical assessment, number (%)	116 (62%) [301]	30 (50%) [60]	0.10
TAPSE (cm), mean (± SD)	20.7 (± 5.1) [160]	18.9 (± 4.5) [39]	0.05
RVS’TDI (cm/s), mean (± SD)	14.1 (± 4.1) [275]	13.1 (± 4.1) [57]	0.10
FAC (%), mean (± SD)	36.5 (± 13.5) [180]	34.3 (± 11.2) [34]	0.37
Basal RV diameter (cm), mean (± SD)	3.8 (± 0.8) [285]	4.0 (± 0.8) [60]	0.07
Dilated basal RV > 4.1 cm, number (%)	84 (30%) [284]	26 (43%) [61]	**0.047**
**Pulmonary pressure**			
Elevated pulmonary pressure, number (%)	93 (33%) [285]	16 (27%) [59]	0.41

Using binary logistic regression, univariable analysis determined white ethnicity, new LV systolic dysfunction, larger LV end-systolic dimension, larger LV end-diastolic dimension and basal RV diameter ≥ 4.1 to be associated with UEA use. When these significant variables were entered into a multivariable analysis, only new LV systolic dysfunction was determined to be independently associated with UEA use with an odds ratio of 2.66, confidence interval 1.2–5.9, p = 0.02. In summary patients undergoing TTE with UEA demonstrated a higher risk group compared with the non-UEA group.

### Safety

Of patients who did not have a TTE with UEA (303), 8 patients (2.6%) died within 24 h of their TTE, 15 (5.0%) within 48 h. Of those who had TTE with UEA (61), 4 (6.6%) died within 24 h of UEA use, 6 (9.8%) within 48 h. There was no significant difference in deaths within 24 h, p = 0.12, or within 48 h, p = 0.14, of TTE with UEA versus without UEA.

## Discussion

With the risk posed to the echocardiographer of becoming infected by COVID-19 as well as that of cross-infection, it is important to establish the efficacy of UEA in this particular patient population to warrant the extra time spent by the patient’s bedside. This study shows the incremental benefit of UEA use, raising the ability to answer the indication for TTE from 75.7 to 88.7%, a relative increase of 17.1%. However, more importantly, this increase in efficacy translates into a change in diagnosis in nearly 40% of patients given UEA. Furthermore, for a test to be useful, it is important to demonstrate that the test resulted in change in management of the patients. This study demonstrates change in management in over a fifth of patients. Clarification of diagnosis is of even greater significance in the context of COVID-19 infection as the subsequent indication for investigations is based on the correct diagnosis, reducing unnecessary tests with the cross-infection risks they entail from transport of the patient to the test facility itself and all the personnel involved.

The clinical utility of UEA in patients with non-diagnostic TTE has been previously demonstrated in both hospitalized and outpatient setting in a large prospective cohort [12]. The greatest benefit was noted in the hospitalized patients. Our study adds to the literature of clinical usefulness of UEA usage in the hospitalized environment but in patients with COVID-19, which is a new infective condition giving rise to a syndrome similar to acute respiratory distress syndrome (ARDS). However, COVID-19 is also characterised by high thromboembolic phenomena, RV dysfunction both as a result of high acute afterload and myocarditis. In addition, LV dysfunction also occurs due to myocarditis and or thromboembolic phenomena affecting the coronary arteries giving rise to acute myocardial infarction. Thus, this study is unique in that it is for the first time we have demonstrated the clinical value of UEA in COVID-19.

The subject of the safe use of UEA in critically ill patients with COVID-19 is also of great importance given that significant proportions of patients admitted to hospital are severely or critically ill, many requiring ITU admission [13]. 43% of patients who had TTE with UEA in this study were on mechanical ventilation on ITU. The safety of UEA in critically ill patients has previously been shown [9, 10] as well as diagnostic benefit of the technique in general [12, 14]. Severe adverse reactions with UEA represent allergic or more commonly pseudo-allergic reactions with anaphylactic or anaphylactoid reactions at the most severe end. The incidence is of the order of 1 in 10,000 [7], generally occurring early following UEA injection.

We therefore looked at the first 48 h post UEA use to assess for associated mortality. Whilst there was no significant difference in mortality at 48 h post TTE with UEA versus without UEA, it is acknowledged that there were few events and therefore, statistical power is limited. Numerically the proportion of patients with 48 h mortality post TTE with UEA was 9.8% versus 5.0% post TTE without UEA. With almost double the incidence, we sought to understand potential contributors to this numerical excess in 48 h mortality. LV systolic dysfunction and larger LV size as well as RV dilatation were associated with the UEA use population. Moreover, there was a trend towards increased prevalence of RV dysfunction and tachycardia in the UEA use population. They are all adverse markers that could contribute to excess mortality. It was also the case that proportionately more patients with UEA were white versus non-white, p = 0.02. This difference may be accounted for by difference in body habitus with less clear windows in the white population. There was a trend toward higher BMI and more respiratory disease in the white population which offers some support for this.

In a preliminary analysis based on 120 hospitalized COVID-19 patients, both LV systolic dysfunction and episodes of tachycardia were associated with mortality [11]. Indeed, the prevalence of higher morbidity in patients undergoing UEA echocardiography in critically ill hospitalized patients has been previously shown. Hospitalized patients undergoing UEA have difficult TTE images due to immobility, hyperinflated lungs due to mechanical ventilation, lung disease, subcutaneous emphysema, surgical incisions, and chest tubes compared to those where TTE images are adequate. These conditions add to the patient morbidity. Large datasets however exist regarding safety in hospitalized patients including patients who are critically ill [7–10].

### Limitations

As a retrospective observational study, though of prospectively assessed patients, it is subject to selection biases which cannot be controlled for. Identification of the need to use UEA as well as the use of UEA itself would have varied between echocardiographer due to differing experience levels as well as the presence of venous access and availability of expertise to gain venous access in such a challenging clinical setting. With a focus on safety, the greatest limitation is that of patient numbers. Whilst 61 out of 364 patients receiving UEA is a reasonable proportion, the low numbers of deaths at 48 h mean that it is difficult to draw statistical conclusions. Greater numbers of patients would be required with an adequate proportion in whom UEA is used to provide statistical certainty as to the safety of UEA. Also, the data presented are from a single centre and therefore may be subject to confounders specific to our patient population which are not then generalisable to the wider population.

## Conclusion

The incremental benefit of UEA use was demonstrated in this study through the significant increase in the ability to answer the questions raised by the referrer, increase in the diagnostic studies and change in management of patients with no significant difference in 24 and 48 h mortality. Patients undergoing UEA are a higher risk population compared with the population undergoing TTE without UEA.

## Data Availability

The final anonymous dataset that supports the findings of this study are available from the corresponding author, RS, upon reasonable request.

## References

[1] Augustine D, Willis J, Robinson S et al (2020) Clinical guidance regarding provision of echocardiography during the COVID-19 pandemic. Br Soc Echocardiogr Published Online First : https://bsecho.org/covid19

[2] Ingram T, Augustine D, Colebourn C et al (2020) COVID-19 clinical guidance. https://bsecho.org/covid19 (accessed 29 Oct 2020)

[3] Skulstad H, Cosyns B, Popescu BA (2020). COVID-19 pandemic and cardiac imaging: EACVI recommendations on precautions, indications, prioritization, and protection for patients and healthcare personnel. Eur Heart J Cardiovasc Imaging.

[4] Senior R, Becher H, Monaghan M et al (2017) Clinical practice of contrast echocardiography: recommendation by the European Association of Cardiovascular Imaging (EACVI) 2017. *Eur Hear J - Cardiovasc Imaging* ;18:1205-1205af. 10.1093/ehjci/jex182 10.1093/ehjci/jex182 28950366

[5] Porter TR, Mulvagh SL, Abdelmoneim SS (2018). Clinical applications of Ultrasonic Enhancing agents in Echocardiography: 2018 American Society of Echocardiography Guidelines Update. J Am Soc Echocardiogr.

[6] Muskula PR, Main ML (2017). Safety with echocardiographic contrast agents. Circ Cardiovasc Imaging.

[7] Wei K, Mulvagh SL, Carson L (2008). The Safety of Definity and Optison for Ultrasound Image Enhancement: a retrospective analysis of 78,383 administered contrast doses. J Am Soc Echocardiogr.

[8] Kusnetzky LL, Khalid A, Khumri TM (2008). Acute Mortality in Hospitalized patients undergoing Echocardiography with and without an ultrasound contrast Agent. Results in 18,671 consecutive studies. J Am Coll Cardiol.

[9] Main ML, Hibberd MG, Ryan A (2014). Acute mortality in critically ill patients undergoing echocardiography with or without an ultrasound contrast agent. JACC Cardiovasc Imaging.

[10] Exuzides A, Main ML, Colby C (2010). A retrospective comparison of mortality in critically ill hospitalized patients undergoing echocardiography with and without an ultrasound contrast agent. JACC Cardiovasc Imaging.

[11] Bioh G, Botrous C, Howard E (2021). Prevalence of cardiac pathology and relation to mortality in a multiethnic population hospitalised with COVID-19. Open Hear.

[12] Kurt M, Shaikh KA, Peterson L (2009). Impact of contrast Echocardiography on evaluation of ventricular function and clinical management in a large prospective cohort. J Am Coll Cardiol.

[13] Liang W, Liang H, Ou L (2020). Development and validation of a clinical risk score to predict the occurrence of critical Illness in hospitalized patients with COVID-19. JAMA Intern Med.

[14] Yong Y, Wu D, Fernandes V (2002). Diagnostic accuracy and cost-effectiveness of contrast echocardiography on evaluation of cardiac function in technically very difficult patients in the intensive care unit. Am J Cardiol.

